# Supplementary material for: A patient-specific lung cancer assembloid model with heterogeneous tumor microenvironments
Source: Nat Commun. 2024 Apr 20;15:3382. doi: 10.1038/s41467-024-47737-z (PMC11032376; doi:10.1038/s41467-024-47737-z)
Supplement: Supplementary file 1 — Supplementary Information [file 41467_2024_47737_MOESM1_ESM.pdf]

Supplementary Information

A patient-specific lung cancer assembloid model with heterogeneous tumor microenvironments

Yanmei Zhang[1,2,3,4, #], Qifan Hu[5, #], Yuquan Pei[6, #], Hao Luo[1,2,3], Zixuan Wang[1,2,3], Xinxin Xu[7], Qing Zhang[4], Jianli Dai[4], Qianqian Wang[4], Zilian Fan[1,2,3], Yongcong Fang[1,2,3], Min Ye[1,2,3], Binhan Li[1,2,3], Mailin Chen[8], Qi Xue[9], Qingfeng Zheng[9], Shulin Zhang[9], Miao Huang[6], Ting Zhang[1,2,3], Jin Gu[5, *], Zhuo Xiong[1,2,3, *]

Supplementary figures

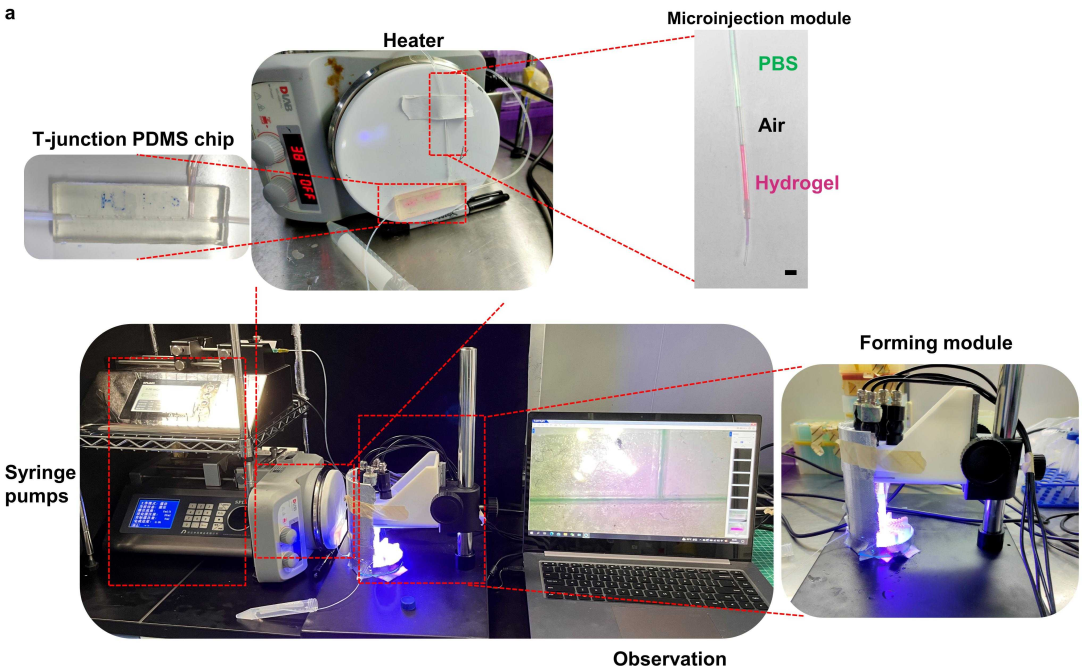

Supplementary Fig. 1 LCA platform setup and the microinjection strategy development, related to Figure 1. a The setup of the droplet-based microfluidic platform. The microfluidic platform mainly consisted of two syringe pumps, a T-junction PDMS chip, a heating module, a lighting module (forming module) and a microscope module (observation). The detail process of LCA platform setup was supplemental in Method Section.

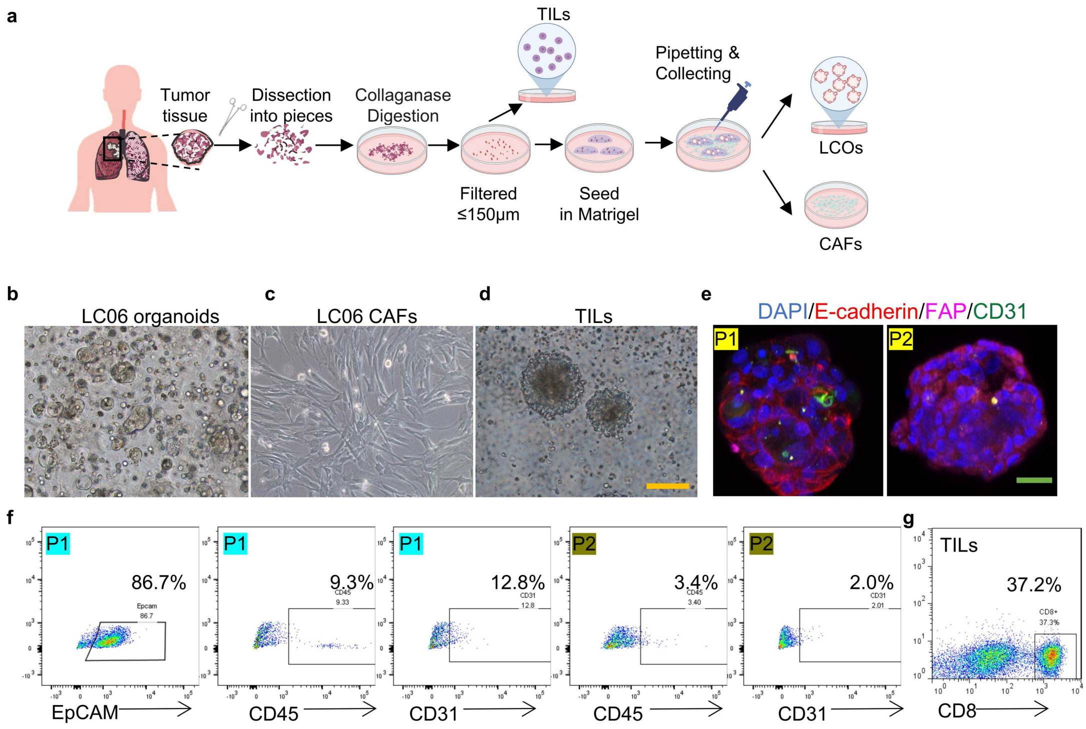

Supplementary Fig. 2 Generation and characterization of lung cancer organoids and TME cells, related to Figure 2. a The schematic illustration of the isolation and culture of TILs, CAFs and LCOs. LCOs, lung cancer organoids; TILs, tumor infiltrating lymphocyte cells. **b,c** Representative images of LC06 LCOs and (c) responding CAFs. **d** Representative images of the immune cells isolated from LC027 tumor. **e** Immunofluorescence staining of human FAP, E-cadherin and CD45 in P1 and P3 tumor organoids of LC06. The experiment was repeated for 3 times. **f** Characterization for LC06 organoids of different passages (P1 and P2) by flow cytometry analysis for the EpCAM+ epithelial cells, CD45+ immune cells and CD31+ endothelial cells. **g** The percentage of CD8+ T cells in TILs of LC27. Scale bar, yellow bar, 200 μm, green bar, 20 μm. For b-c, the experiments were repeated in 35 patients.

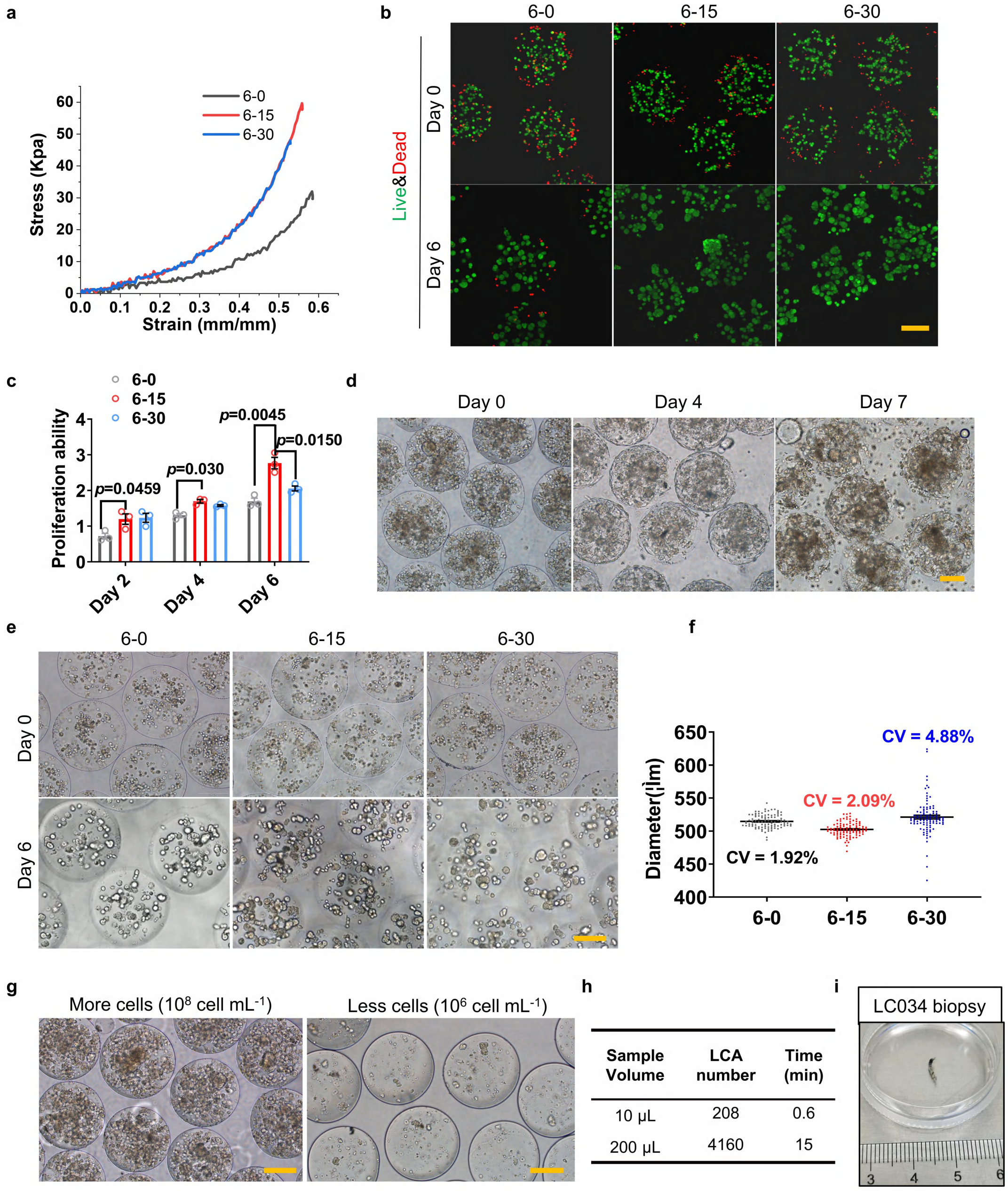

Supplementary Fig. 3 Establishment of uniform LCAs using GelMA-Matrigel hydrogel through a microfluidics-based microinjection strategy, related to Figure 2. a Representative stress-strain curves of GelMA-Matrigel hydrogels. **b** Live & dead staining for the A549 cell-laden microgels at day 0 and day 6 post fabrication. **c** The normalized proliferation ability of cells in A549 cancer cell-laden microgels of each group (*n* = 3 biologically independent samples). **d** Representative images of LC05 LCAs at day 0, 4 and 7 after fabrication. **e** Representative bright-field images of cancer cell-laden microgels at day 0 and day 6. **f** Uniformity analysis for microgel diameters of 6-0, 6-15 and 6-30 groups (*n* = 100 independent cell-laden microgels). **g** Representative images of LCAs with high cell density (10^8 mL^{-1}) and low cell density (10^6 mL^{-1}). **h** LCA numbers and time costs resulting from the use of different sample volumes. **i** Image of the needle biopsy from lung tumor. Scale bar, yellow bar, 200 μm; two-sided Student's t test is used, data is presented as mean ± S.E.M. For b,d,e, g,

each experiment was repeated independently with similar results for 3 times. Source data are provided as a Source Data file.

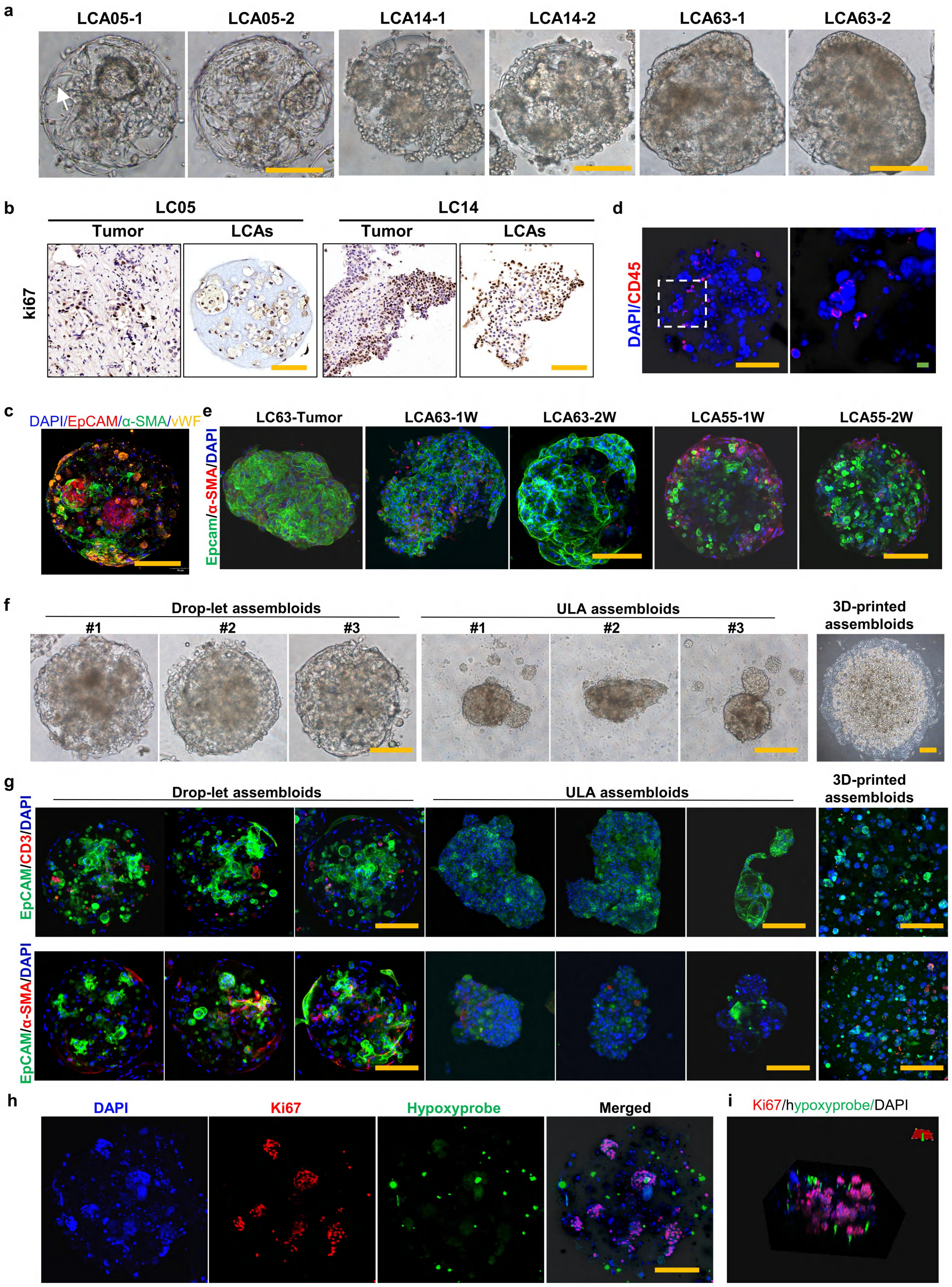

Supplementary Fig. 4 LCAs recapitulate the histologic and TME features of parental tumors, related to Figure 3. a Representative bright field microscopy images of LCAs derived from LC05, LC14 and LC63 patients. The experiment was repeated in 35 patient samples. **b** Sample images of immunohistochemistry staining for Ki67. **c** Representative immunofluorescence staining image of human EpCAM, α-SMA and endothelial marker vWF in LCAs. **d** Immunofluorescence staining of human CD45 in LC27 LCAs. **e** Immunofluorescence staining of human α-SMA and

EpCAM in tumor fragments and corresponding LCAs cultured for 1 week and 2 weeks. **f** Representative bright-field images of the LCAs generated via droplet microfluidic, low-attachment U-plate method and 3D bioprinting. **g** Immunofluorescence staining of human α-SMA, EpCAM and CD3 in LCAs generated via droplet microfluidic, low-attachment U-plate method and 3D bioprinting. **h,i** Representative immunofluorescence images and 3D image (i) showing the proliferative marker Ki-67 and hypoxia marker pimonidazole in LC27 LCAs. Scale bar, yellow bar, 200 μm, green bar, 20 μm. For b-h, each experiment was repeated independently for 3 times with similar results.

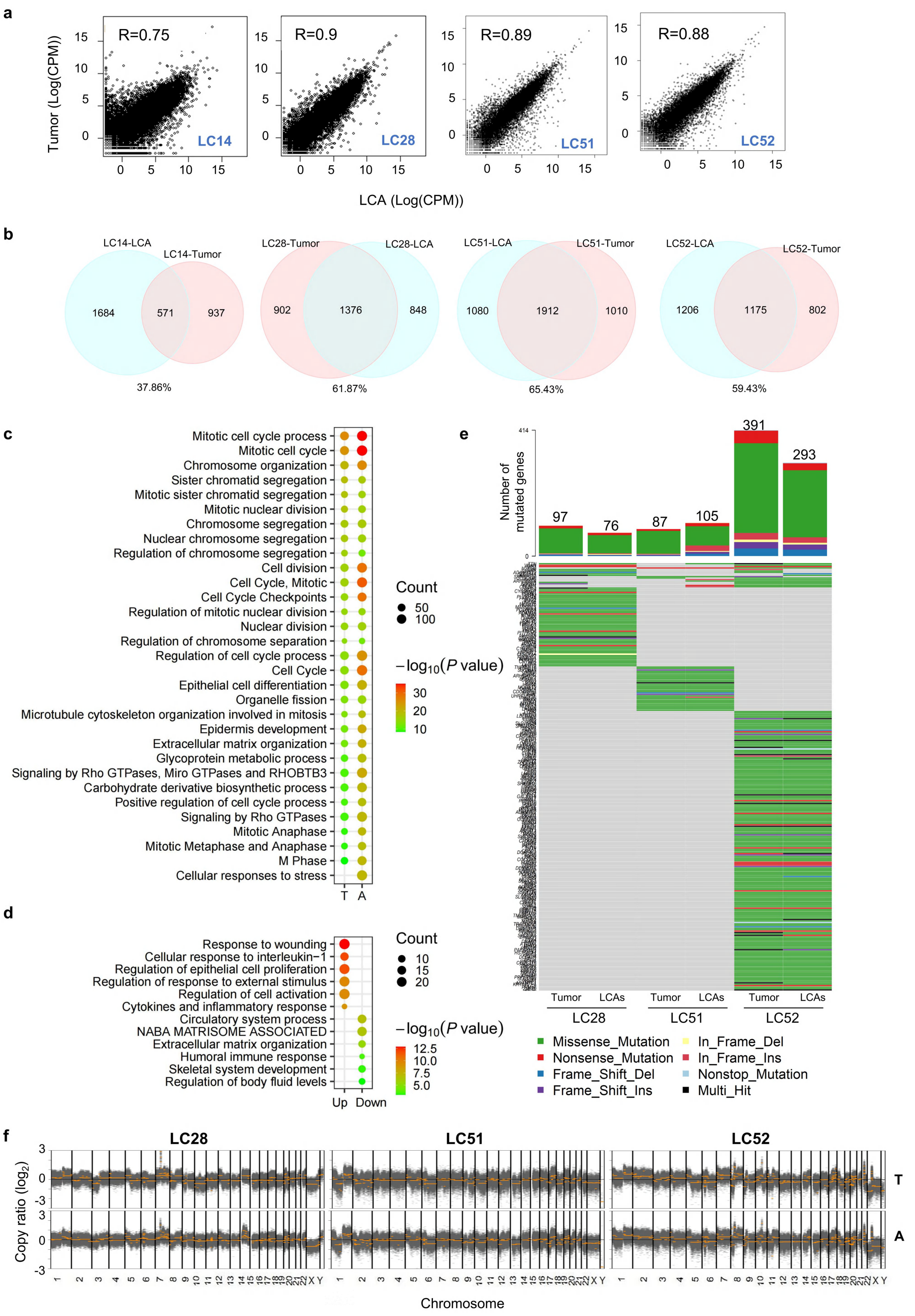

Supplementary Fig. 5 Bulk RNA-Seq and exome sequencing analysis of parental tumors and corresponding LCAs, related to Figure 4. a Scatterplots comparing gene expression in parental tumors and corresponding LCAs. R, Pearson correlation coefficients. **b** Venn diagrams showing the overlap of upregulated genes between the

LCAs and the original tumors compared to the corresponding normal tissues. **c** The comparison of the top 20 pathways enriched in both LCAs and parent tumors. (T, tumor; A, LCA). **d** Bubble plots of enriched gene ontology biological process terms in the down- and upregulated genes in all LCAs compared to all parental tumors. **e** The histogram at top showing the number of genetic alterations observed in each parental tumor and corresponding LCA. The lower heat map showing the mutations in LCAs and corresponding tumors of each patient. **f** Sample copy ratio tracks of parental tumors and corresponding LCAs. Ratio was normalized to the corresponding normal tissue.

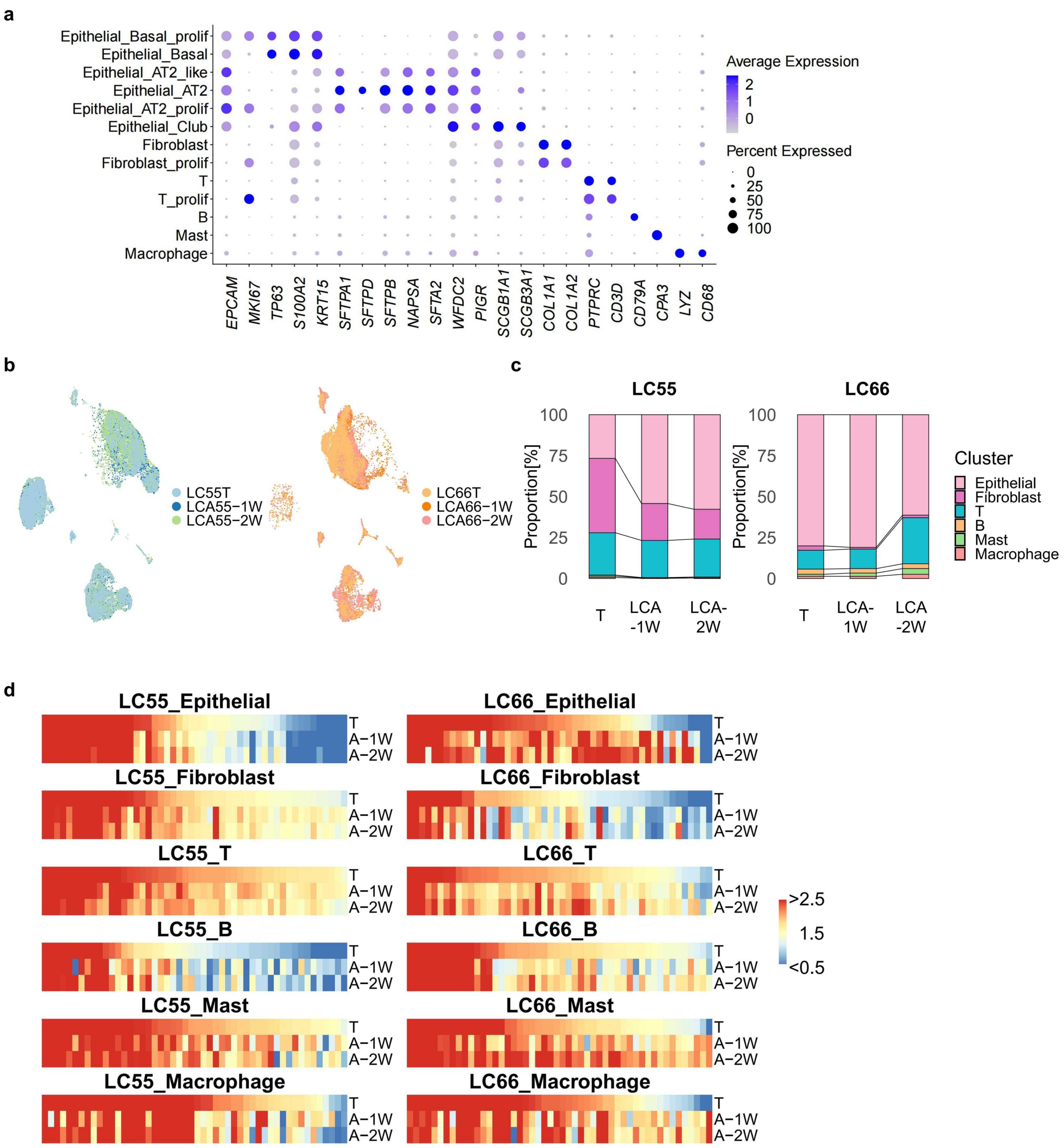

Supplementary Fig. 6 Single-Cell RNA-Seq analyses of parental tumors and corresponding LCAs, related to Figure 5. a Dot plot showing the marker genes of each cell type cluster defined in Fig. 5c. **b** UMAP plot showing the sample types (parental tumors and corresponding LCAs cultured for 1 week and 2 weeks) from two sample sources (LC55 and LC66). **c** Comparative analysis between primary tumors (LC55 and LC66) and corresponding LCAs for the proportions of individual cell types. **d** Heatmap showing the expression levels of cell type-specific top 50 genes in 3 sample types (tumors and corresponding LCAs cultured for 1 week and 2 weeks). T, tumor; A-1w, LCAs cultured for 1 week.

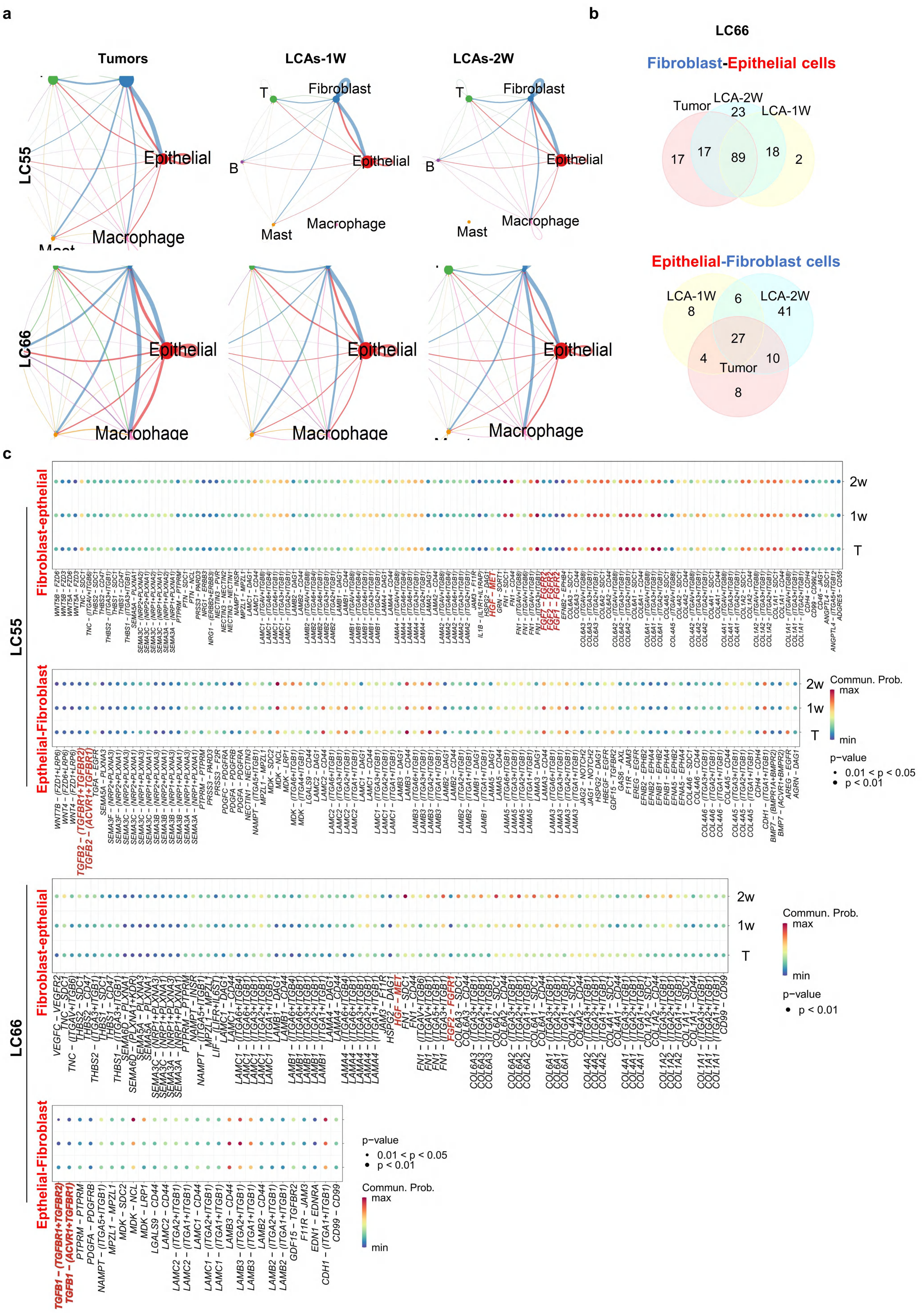

Supplementary Figure 7. LCAs maintained the cell–cell interactions of parental tumors, related to Figure 5. a Circle plots of cell-cell communication network of 6 samples. The width of edges represents the strength of the communication. **b** Venn diagram showing the overlap in ligand - receptor pairs between CAFs and tumor

epithelial cells of LCAs and the parental tumor tissues of LC55 and LC66 patients.
c Bubble plots showing the shared significant ligand - receptor pairs from CAFs to epithelial cells and from epithelial to CAFs in LCAs and parent tumors. The red color marked the key ligand - receptor interactions of interest.

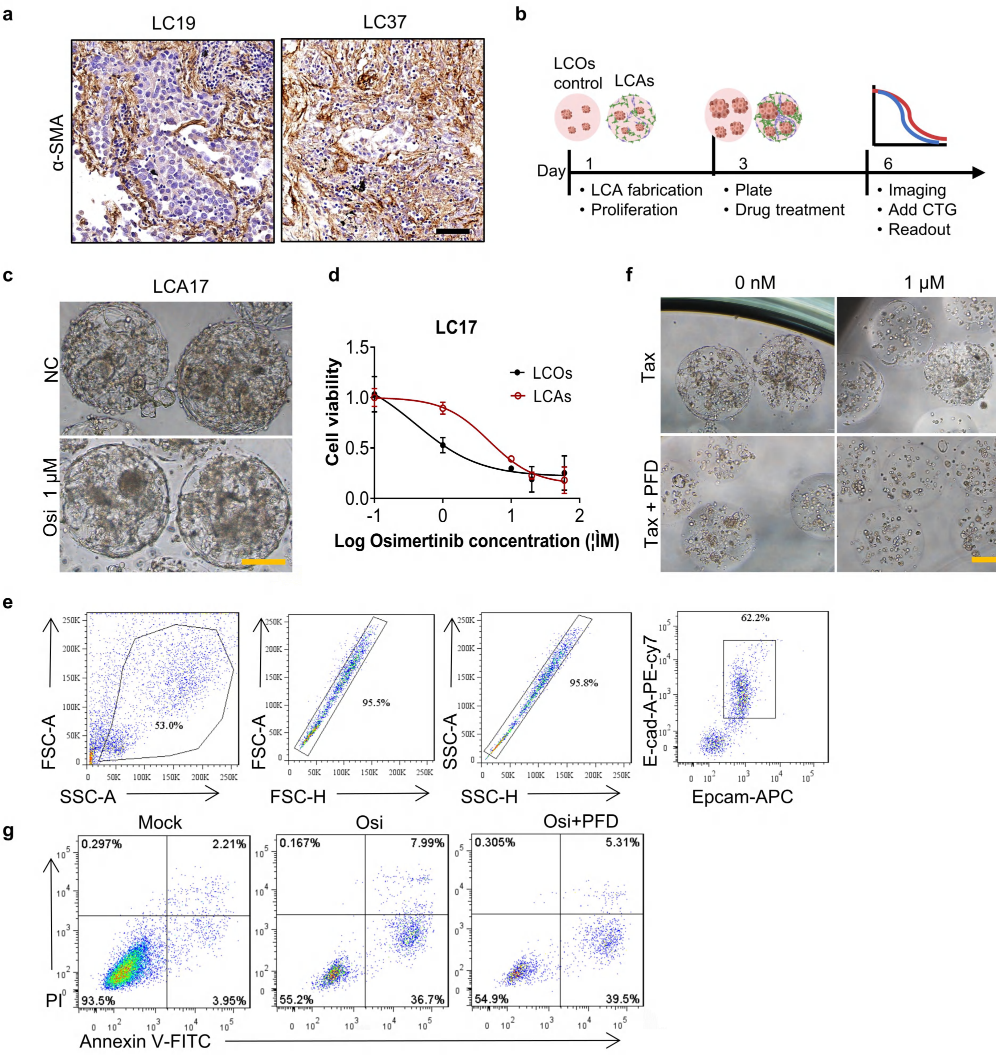

Supplementary Fig. 8 Targeted drug treatment based on heterogeneous CAFs using LCA platform, related to Figure 6. a IHC staining for α-SMA+ CAFs for the tumors derived from different patients. **b** Schematic overview of the drug-screening protocol with LCAs. **c** Representative images of LCA17 treated with 1 μM Osimertinib for 3 days. **d** Dose–response curves of LC17 assembloids after 3 days of treatment with Osimertinib treatment. (*n* = 3 biologically independent samples) **e** The gating strategy of flow cytometry analysis for tumor epithelial cell apoptosis. **f** Representative images of LC23 LCAs treated with Taxol and 2 μM PFD for 3 days.
g Apoptosis analysis by flow cytometry for EpCAM+ tumor cells in LC23 LCAs with the combined drug treatment. Scale bar, black bar, 50 μm, yellow bar, 200 μm. Data are presented as mean ± SEM. Source data are provided as a Source Data file.

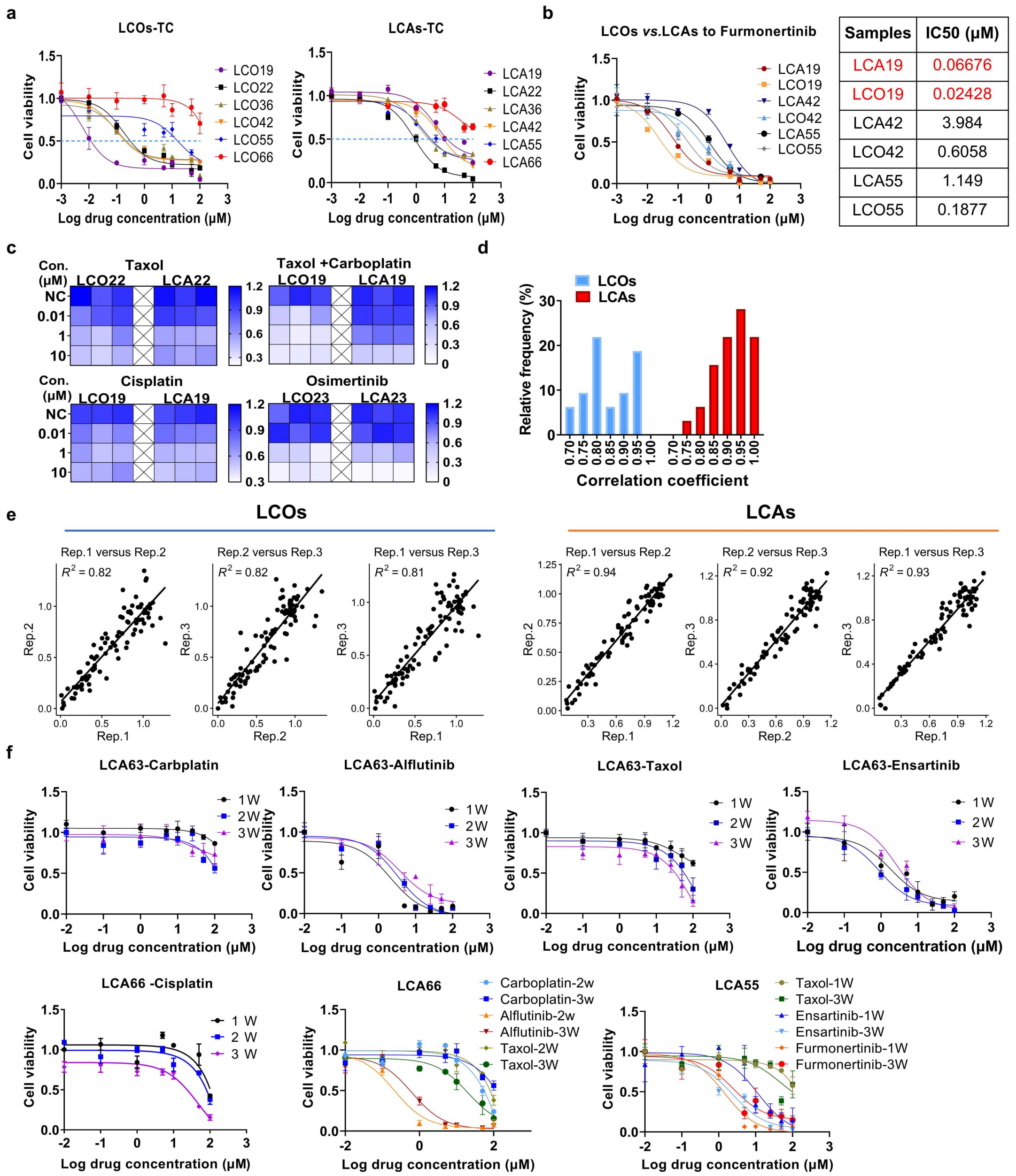

Supplementary Fig. 9 LCAs as a powerful preclinical model for personalized drug testing, related to Figure 7. **a** The fitted dose–response curves illustrate the responses of the LCAs and LCOs to Taxol + carboplatin (TC) (n = 3 biologically independent samples). **b** Comparison of responses to EGFR targeted drug (Furmonertinib) between LCOs and corresponding LCAs derived from 3 patients. (LC19, EGFR-mutant patient, LC42 and LC55 had no EGFR mutation) (n = 3 biologically independent samples). **c** Heat map summarizing the results of the drug testing assays. **d** The distribution of the Pearson correlation coefficients of two results in three parallel drug sensitivity tests (n = 29 drug-sample pairs). **e** Linear correlation fittings between any two sets of parallel experiments of drug testing assays in LCOs and LCAs. Rep., replication. **f** Dose-response curves of tumor assembloids cultured for different time. (n = 3 biologically independent samples). Data are presented as mean ± SEM. Source data are provided as a Source Data file.

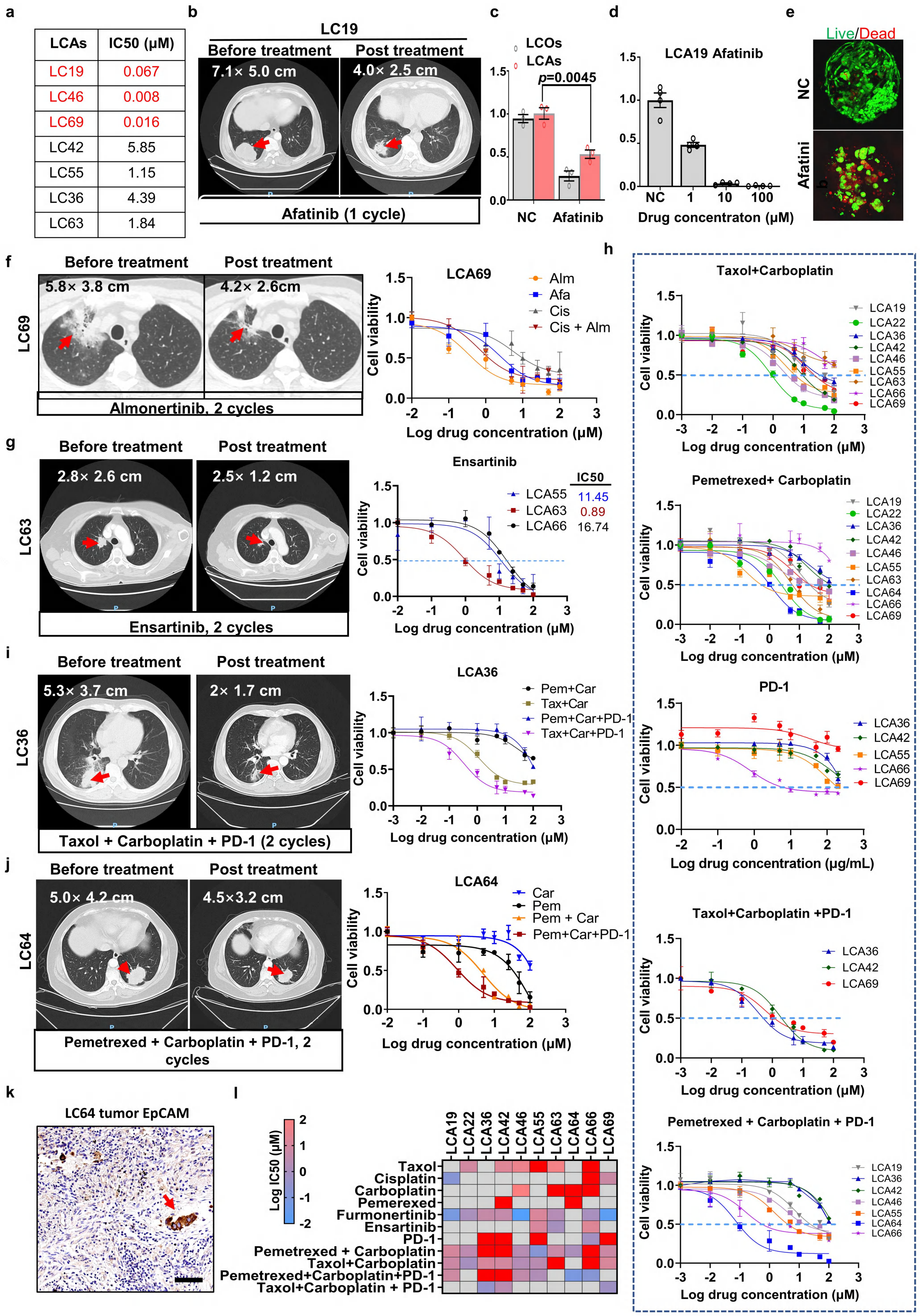

Supplementary Fig. 10 LCAs as a powerful preclinical model for personalized drug testing, related to Figure 7. a The Furmonertinib IC50 values listed in the table were interpolated from the fitted dose–response curves of Figure 7a. R means IC50 is not available since the viability of the LCO is >50% under all the concentrations. **b**

CT images of patient LC19 before and after neoadjuvant therapy. The size of the lung tumor, indicated by red arrow, decreased from 7.1×5.0 cm^2 to 4.0×2.5 cm^2 after 1 cycle of Afatinib treatment. **c** The drug-response profile of LCAs and LCOs from LC19 (*n* = 3 biologically independent samples). **d** The Afatinib-response profile of LCAs from patient LC19 with EGFR mutation. (*n* = 3 biologically independent samples). **e** The live/dead staining images showed drug-response of LCAs from patient LC19. NC, negative control. (*n* = 3 independent experiments). **f,g** The PET-CT images (left) and dose–response curves of LCAs of patient LC69 and LC63 (*n* = 3 biologically independent samples). **h** The fitted dose–response curves of LCAs of different patients exposed to carboplatin-based chemotherapy drugs, immune checkpoint blockade (PD-1) and PD-1 combined with carboplatin-based chemotherapy drugs. **i,j** PET-CT images (left) and dose–response curves (right) of LCAs of patient LC36 and LC64 with PD-1 plus Carboplatin-based chemotherapy (*n* = 3 biologically independent samples). **k** IHC staining for the EpCAM+ epithelial cancer cells in tumor of LC64 indicated by the red arrow. The experiment was repeated for 3 times with similar results. **l** Heat map illustrating the sensitivities of 10 LCA lines to chemotherapies, targeted therapies and PD-1 combined with chemotherapy. The sensitivity is denoted by the log10(IC50(μM)) value. Two-sided Student’s t test is used. Data are presented as mean ± SEM. Scale bar, black bar, 50μm. Source data are provided as a Source Data file.

Supplementary Table 1. Comparison of LCOs and LCAs

	LCOs	LCAs
Morphology	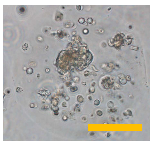	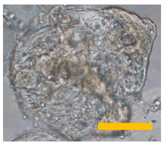
TME maintenance	DAPI/EpCAM/α-SMA 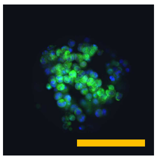	DAPI/EpCAM/α-SMA 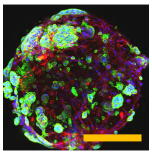
Reproducibility of drug testing (Mean Pearson correlation)	0.74	0.89
Drug responses	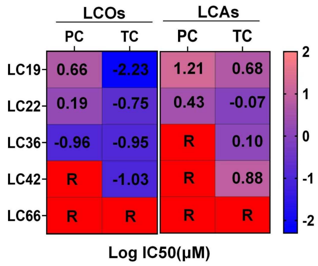	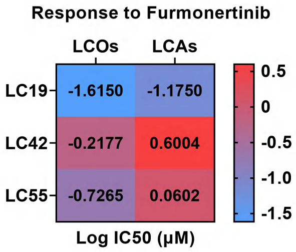

Scale bar: 200 μm; PC, pemetrexed + carboplatin; TC, taxol + carboplatin.

Supplementary Table 2. Pathological information of the clinical samples

Case No.	Patient ID	Histology	TNM Stage	Differentiation	Treatment	Mutation	TPS (%)	LCO or not	CAFs	LCA or not
1	hLC05	AC	T2N0M0	Moderate-poor	None	EGFR 19	/	Yes	Yes	Yes
2	hLC06	AC	T1N0M0	Poor	None	EGFR 19	/	Yes	Yes	Yes
3	hLC07	SqCC	T2N0M0	Moderate-poor	None	/	/	Yes	Yes	Yes
4	hLC12	AC	pT1cN0	Moderate	None	EGFR 21	0	/	/	Yes (direct)
5	hLC13	SqCC	ypT3bN0	Moderate	Abraxane + Cisplatin + Toripalimab	ALK	85	/	/	Yes(direct)
6	hLC14	AC	pT2aN1	Moderate-poor	None	EGFR 21	0	Yes	Yes	Yes
7	hLC15	AC	ypT2bN1	Moderate	None	EGFR 21	60	/	/	fialed（Direct）
8	hLC16	AC	pT1cN0	Moderate	Pemetrexed + Carboplatin	EGFR	90	Yes	Yes	Yes
9	hLC17	AC	pT1miNo	Moderate-poor	None	EGFR 21	0	Yes	Yes	Yes
10	hLC18	AC	YpT0N0	Well	Pemetrexed + cisplatin + PD-1	/	/	Yes	No	/
11	hLC19	AC	ypT2bN1	Moderate	Afatinib	EGFR 19	/	Yes	Yes	Yes
12	hLC20	SqCC	pT2bNO	Moderate/Poor	None	/	7	Yes	No	/
13	hLC21	AC	cT2aNOMO IB	Moderate	None	EGFR21	0	Yes	Yes	Yes
14	hLC22	AC	pT2aN1	Poor	None	/	0	Yes	Yes	Yes
15	hLC23	AC	pT1cN0	Moderate	None	EGFR 19	0	Yes	Yes	Yes
16	hLC24	AC	pT1bN0M0	Moderate	None	EGFR 19	1	Yes	Yes	/
17	hLC25	AC	pT1cN0M0	Moderate	None	EGFR 21	1	No	/	/
18	hLC26	AC	pT1cN0M0	Moderate-poor	None	/	10	Yes	Yes	/
19	hLC27	AC	pT1cN0M0	Moderate	None	/	5	Yes	Yes	Yes
20	hLC28	AC	pT1cN1M0	Poor	None	/	8	Yes	Yes	Yes
21	hLC29	AC	pT1miN0M0	Well	None	/	/	/	/	/
22	hLC30	AC	pT2aN0M0	/	Radiofrequency ablation	/	1	/	/	/
23	hLC31	AC	pT1bN0M0	Moderate	None	/	/	/	/	/
24	hLC32	AC	ypT2aN0M0	/	Cisplatin + Gefitinib	EGFR 19	0	/	/	/
25	hLC33	AC	pT1miN0M0	Moderate	None	/	/	/	/	Yes
26	hLC34	AC	pT2N0M0	Poor	None	/	3	/	/	Yes
27	hLC35	AC	pT1aN0M0	/	None	/	/	Yes	Yes	Yes
28	hLC36	AC	T2N2M0	/	Taxol + carboplatin + PD-1	No	1	Yes	Yes	Yes
29	hLC38	SqCC	pT1cN0M0	Poor	None	/	0	Yes	Yes	Yes
30	hLC39	AC	pT1bN0M0	Well	None	/	/	Yes	No	/
31	hLC40	AC	pT1cN0M0	/	Pemetrexed + cisplatin + PD-1	EGFR 21	0	/	/	/
32	hLC41	AC	pT2aN0M0 IB	/	None	KRAS G12C	/	Yes	Yes	Yes
33	hLC42	AC	cT2bN1M0	Poor	Taxol + Carboplatin + PD-1	No	6	Yes	Yes	Yes
34	hLC45	SqCC	/	Moderate	None	/	/	Yes	Yes	Yes
35	hLC46	SqCC	/	/	Taxol + carboplatin + Furmonertinib	EGFR 20	/	Yes	Yes	Yes
36	hLC51	AC	pT2aN1M0	Moderate-poor	Alectinib	ALK(+)	65	Yes	Yes	Yes
37	hLC52	AC	/	Poor	None	/	＜1	Yes	Yes	Yes
38	hLC54	AC	ypT2aN0	Moderate	Furmonertinib	EGFR 21	0	Yes	Yes	Yes
39	hLC55	AC	cT1N0M0	Moderate	None	KRAS 2	0	Yes	Yes	Yes
40	hLC62	AC	pT1cN0	Moderate	None	/	/	Yes	Yes	Yes
41	hLC63	AC	ypT1cN1M0	/	Ensartinib	ALK(+)	/	Yes	Yes	Yes
42	hLC64	AC	ypT2bN2	Poor	Pemetrexed + Carboplatin + PD-1	ERBB 2	/	Yes	Yes	Yes
43	hLC65	AC	ypT0N1	/	Pemetrexed + Carboplatin + PD-1	/	/	Yes	Yes	/
44	hLC66	AC	pT2aN0	Poor	None	KRAS 2	10	Yes	Yes	Yes
45	hLC69	AC	/	/	Almonetinib	EGFR 19	2	Yes	Yes	Yes
46	hLC70	AC	/	Moderate	None	EGFR 19	/	Yes	Yes	/
47	hLC72	AC	cT1bN0M0	Moderate	None	EGFR 21	/	Yes	Yes	Yes
48	hLC74	AC	pT2bNO	Poor	None	/	2	Yes	Yes	Yes

AC, adenocarcinoma; SqCC, squamous cell carcinoma; /, no analysis ; TPS, tumor cell Proportion Score for PD-L1 expression.

SupplementaryTable 3. Drug treatment of samples used for clinical drug response consistency studies

Patient ID	Mutation	Sample type	Clinical Drug Treatment	Clinical Responses （PET-CT）
hLC19	EGFR 19	Surgical tumor	Afatinib	PR
hLC36	None	Surgical tumor	Taxol + carboplatin + PD-1	PR
hLC42	None	Surgical tumor	Taxol + Carboplatin + PD-1	PR
hLC46	EGFR 20	Tumor biopsies	Taxol + carboplatin + Furmonertinib	PR
hLC63	ALK(+)	Surgical tumor	Ensartinib	PR
hLC64	None	Surgical tumor	Pemetrexed + Carboplatin + PD-1	SD
hLC69	EGFR 19	Surgical tumor	Almonetinib	PR

SupplementaryTable 4. Chemotherapy and targeted therapy drugs

Chemicals	Source	Catalog NO.
Afatinib	Selleckchem	S1011
Furmonertinib	TargetMol	T7175
Almonertinib	Selleckchem	S8817
Carboplatin	Selleckchem	S1215
Cisplatin	Selleckchem	S1166
Ensartinib	Selleckchem	S2934
Osimertinib	Selleckchem	S5078
Taxol	Selleckchem	S1150
Pemetrexed	Selleckchem	S1135
Pirfenidone	TargetMol	T2386
Salinomycin	Selleckchem	S8129

SupplementaryTable 5. The recipe of LCO medium

Regents for organoid culture	Source	Catalog No.	Final concentration
AD-DMEM/F12	ThermoFisher Scientific	11320-033	
GlutaMAX	Gibco	35050-061	1%(v/v)
HEPES	Gibco	15630-080	1%(v/v)
Penicillin/Streptomycin	Hyclone	SV30010	1%(v/v)
B-27 Supplement (50X)	Invitrogen	17504044	1 X
N2 Supplement (100X)	Invitrogen	17504048	1 X
EGF	Novoprotein	C029	40 ng mL-1
Human FGF-basic	Novoprotein	C751	10 ng mL-1
NEAA	Gibco	11140050	1%(v/v)
Wnt3A	Novoprotein	C06D	50 ng mL-1
Noggin	Novoprotein	CB89	50 ng mL-1
ROCK inhibitor	Selleck	S1049	10 μM
A83-01	Selleck	S7692	0.5 μM
SB202190	Selleck	S1077	3 μM

SupplementaryTable 6. Reagents and critical kits

Regents or Resources	Source	Catalog No.
Mineral oil	Sigma-Aldrich	M8410
Span 80	Sigma-Aldrich	S6760
DMEM	Thermo Fisher Scientific	C11995500BT
RPMI 1640	Sigma-Aldrich	R8758
Phosphate Buffered Saline (1x)	HyClone	SH30256.01
FBS	BI	04-001-1ACS
Bovine Serum Albumin	Sigma-Aldrich	R00912
IL-2	Novoprotein	GMP-CD66
0.25% Trypsin-EDTA (1x)	Gibco	25200-056
Calcein-AM/PI kit	Dojindo	C542
Collagenase I	Wako	17100-017
Collagenase IV	Wako	17104-019
CellTiter-Glo 3D cell viability assay	Promega	G9683
Cell Counting Kit-8	Dojindo	CK04
PBS	Gibco	C3580-0500
GelMA	EFL	GM-60
Matrigel	Corning BD	354230
4%PFA	LEAGENE	76104
Tween-20	Beyotime	ST825
1X RBC lysis buffer	Thermo	00433357
Triton X-100	Sigma-Aldrich	Cat# T9284
Hypoxyprobe	Abcam	HP6-100Kit
Alexa Fluor® 594-AffiniPure Goat	Abcam	ab150116
DAPI	Solarbio	C0060
FGF7 Human ELISA Kit	PYRAM	CEK1155
HGF Human ELISA Kit	Solarbio	SEKH-0201
Tislelizumab (PD-1)	BeiGene	BGB-A317
DMSO	Sigma-Aldrich	Cat# D2650

SupplementaryTable 7. Antibody information

Antibodies	Source	Catalog No.	Dilution
Rabbit monoclonal anti-EpCAM [EPR20532-225]	Abcam	ab223582	1:1000
Sheep polyclonal anti-vWF	Abcam	ab11713	1:600
Mouse anti-alpha-smooth muscle anctin antibody [1A4]	Abcam	ab7817	1:800
Mouse monoclonal anti-CD45 [HI30]	Biolegend	304002	1:50
Rabbit monoclonal CD3 antibody [SP162]	Abcam	ab135372	1:200
FITC anti-human CD8a Antibody [RPA-T8]	Biolegend	301006	1:20
Rabbit monoclonal anti-Ki67 [EPR3610]	Abcam	ab216709	1:1000
FITC anti-human CD31 Antibody [WM59]	Biolegend	303104	1:20
Mouse monoclonal anti-E-cadherin [M168]	Abcam	ab76055	1:200
Rabbit monoclonal anti-FAP [E1V9V]	CST	66562	1:200
Rabbit monoclonal anti-CK7[UMAB161]	ZSGB-BIO	ZM-0071	Working fluid
Alexa Fluor 647-AffiniPure Donkey Anti-Sheep IgG (H+L)	Jackson	713-605-003	1:500
Alexa Fluor 488-AffiniPure Donkey Anti-rabbit IgG (H+L)	Jackson	JAC-111-545-003	1:500
Alexa Fluor® 594-AffiniPure Goat Anti-Rabbit IgG (H+L)	Jackson	JAC-111-585-144	1:500
Alexa Fluor® 594-AffiniPure Goat Anti-mouse IgG (H+L)	Abcam	ab150116	1:500
