# Supplementary material for: A patient-specific lung cancer assembloid model with heterogeneous tumor microenvironments
Source: Nat Commun. 2024 Apr 20;15:3382. doi: 10.1038/s41467-024-47737-z (PMC11032376; doi:10.1038/s41467-024-47737-z)
Supplement: Supplementary file 3 — Description of Additional Supplementary Files [file 41467_2024_47737_MOESM3_ESM.pdf]

Description of Additional Supplementary Files

Supplementary Datasets

Supplementary Data 1. Comparasion of high-resolution H&E staining images between LCAs and their corresponding parental tumors, related to Figure 3a.
