# Supplementary material for: A patient-specific lung cancer assembloid model with heterogeneous tumor microenvironments
Source: Nat Commun. 2024 Apr 20;15:3382. doi: 10.1038/s41467-024-47737-z (PMC11032376; doi:10.1038/s41467-024-47737-z)
Supplement: Supplementary file 4 — Supplementary Data 1 [file 41467_2024_47737_MOESM4_ESM.pptx]

## Slide 1
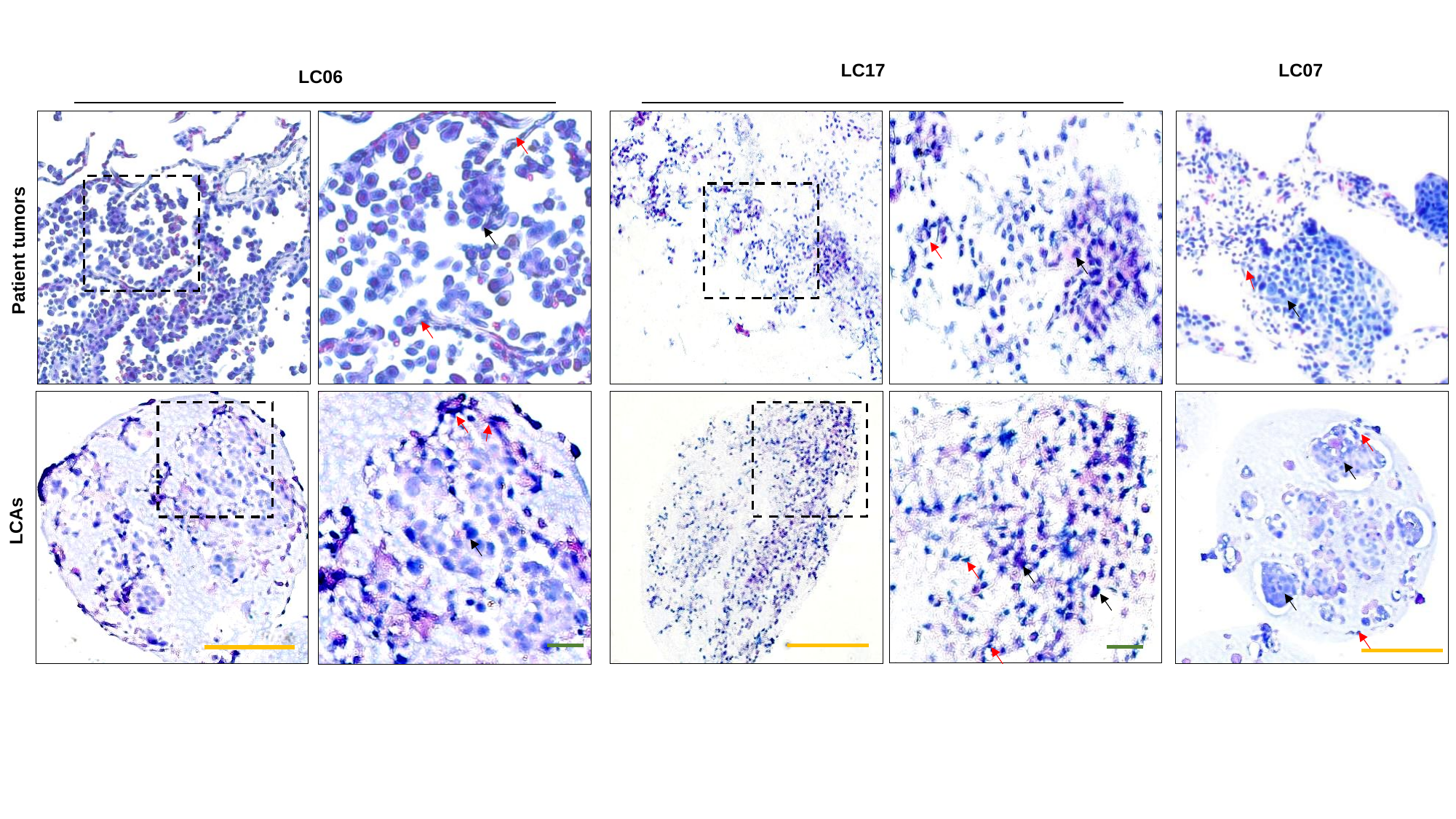

LC17
LC07
LC06
Patient tumors
LCAs

## Slide 2
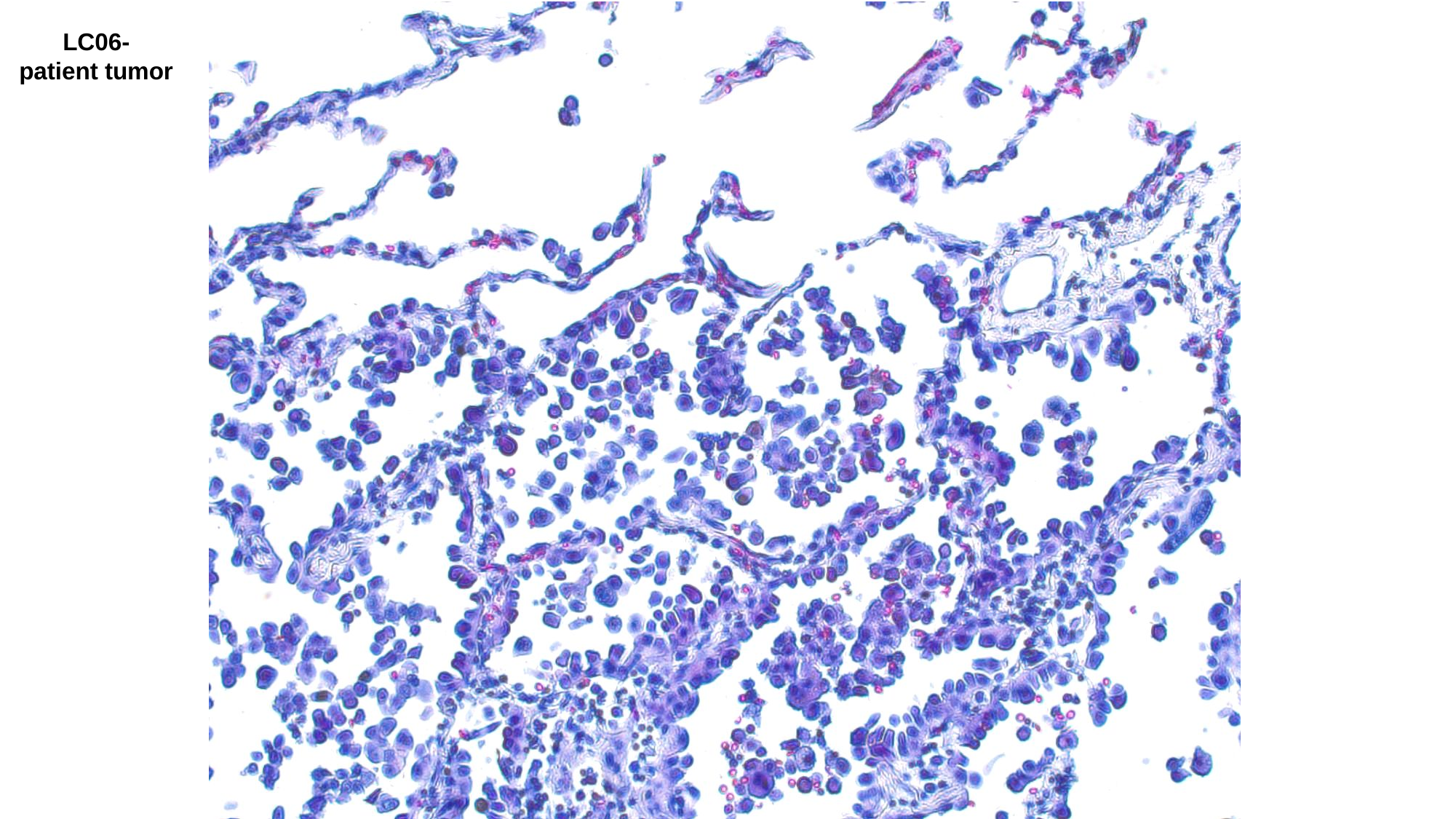

LC06-
patient tumor

## Slide 3
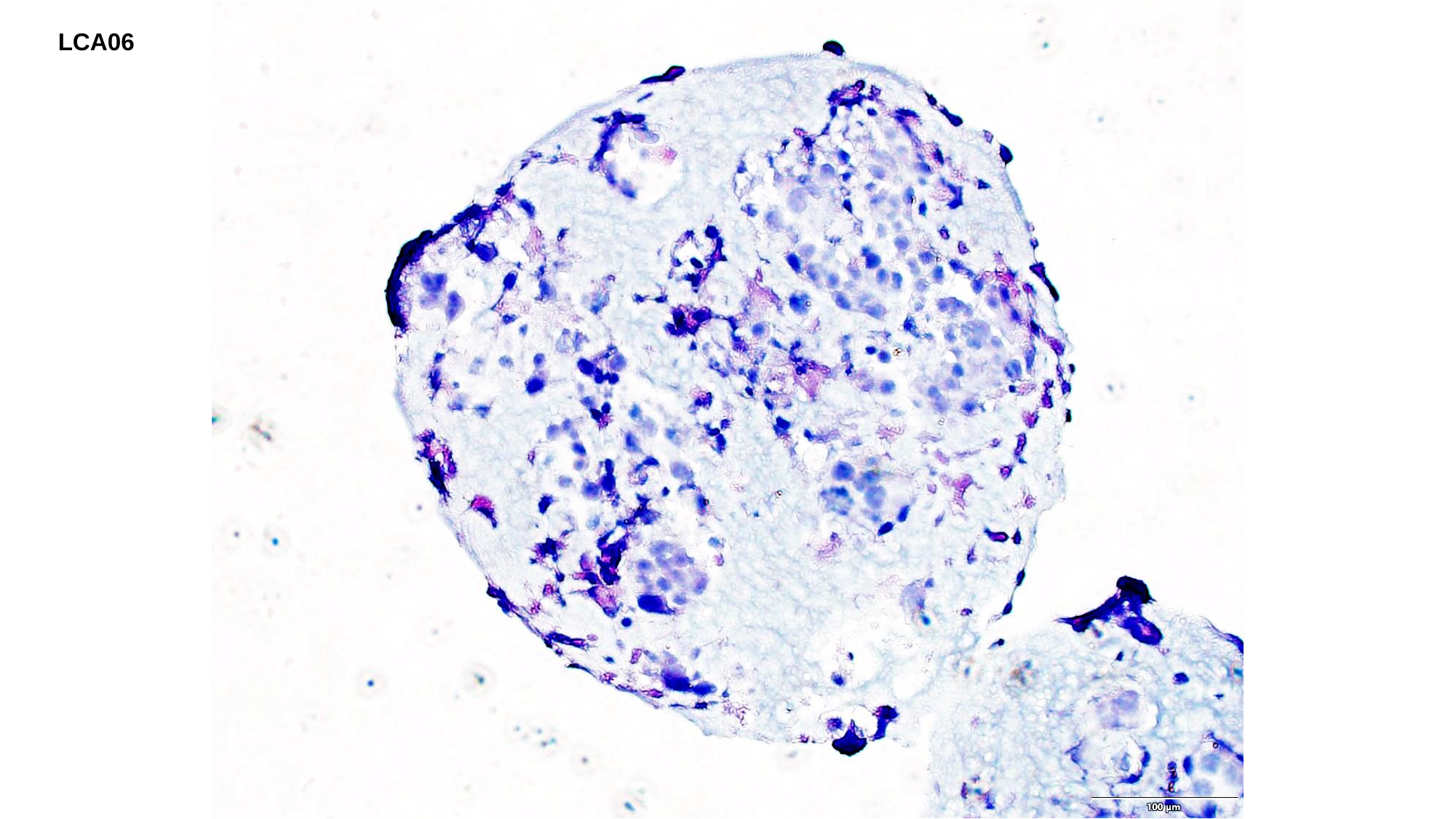

LCA06

## Slide 4
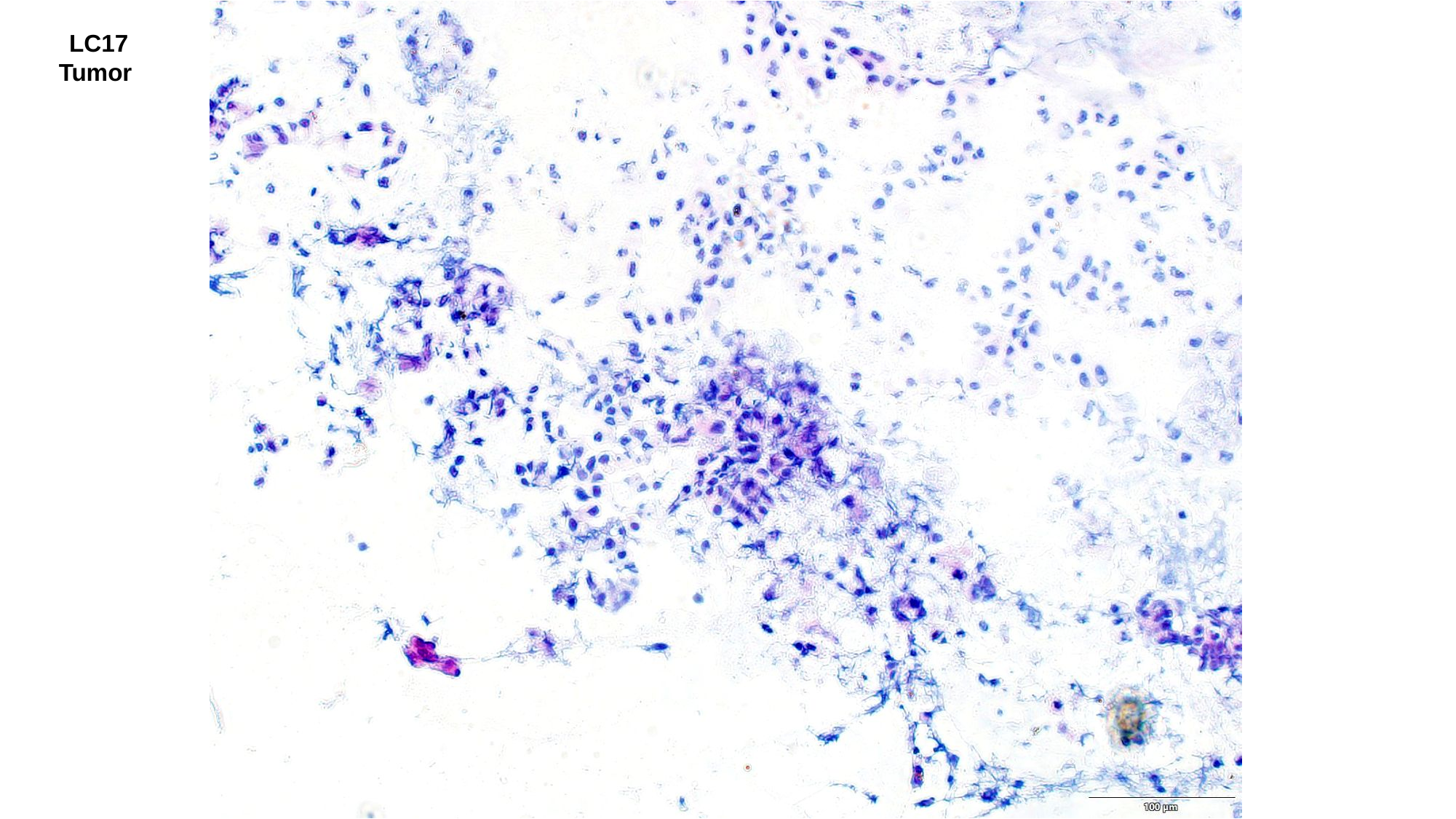

LC17
Tumor

## Slide 5
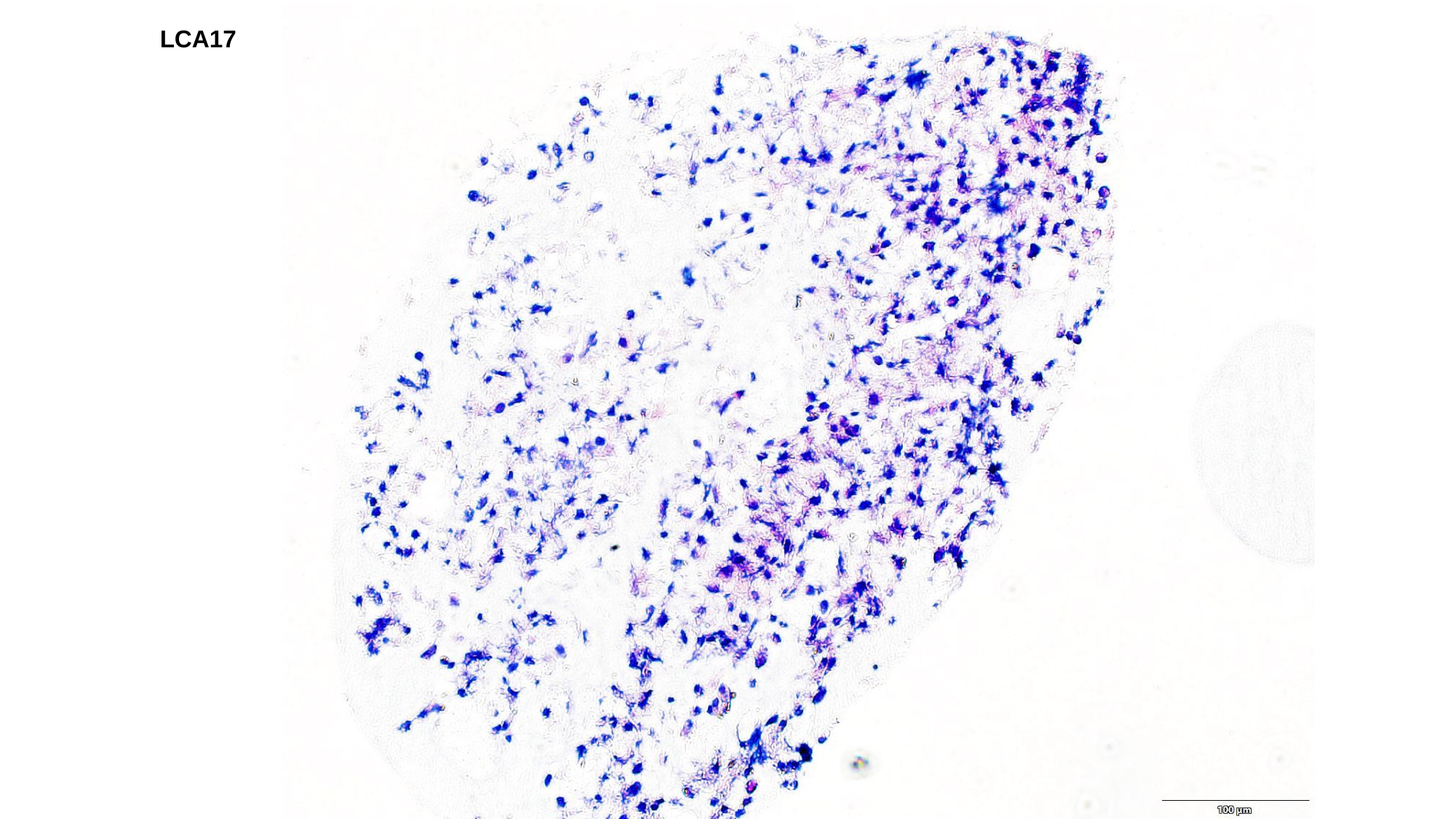

LCA17

## Slide 6
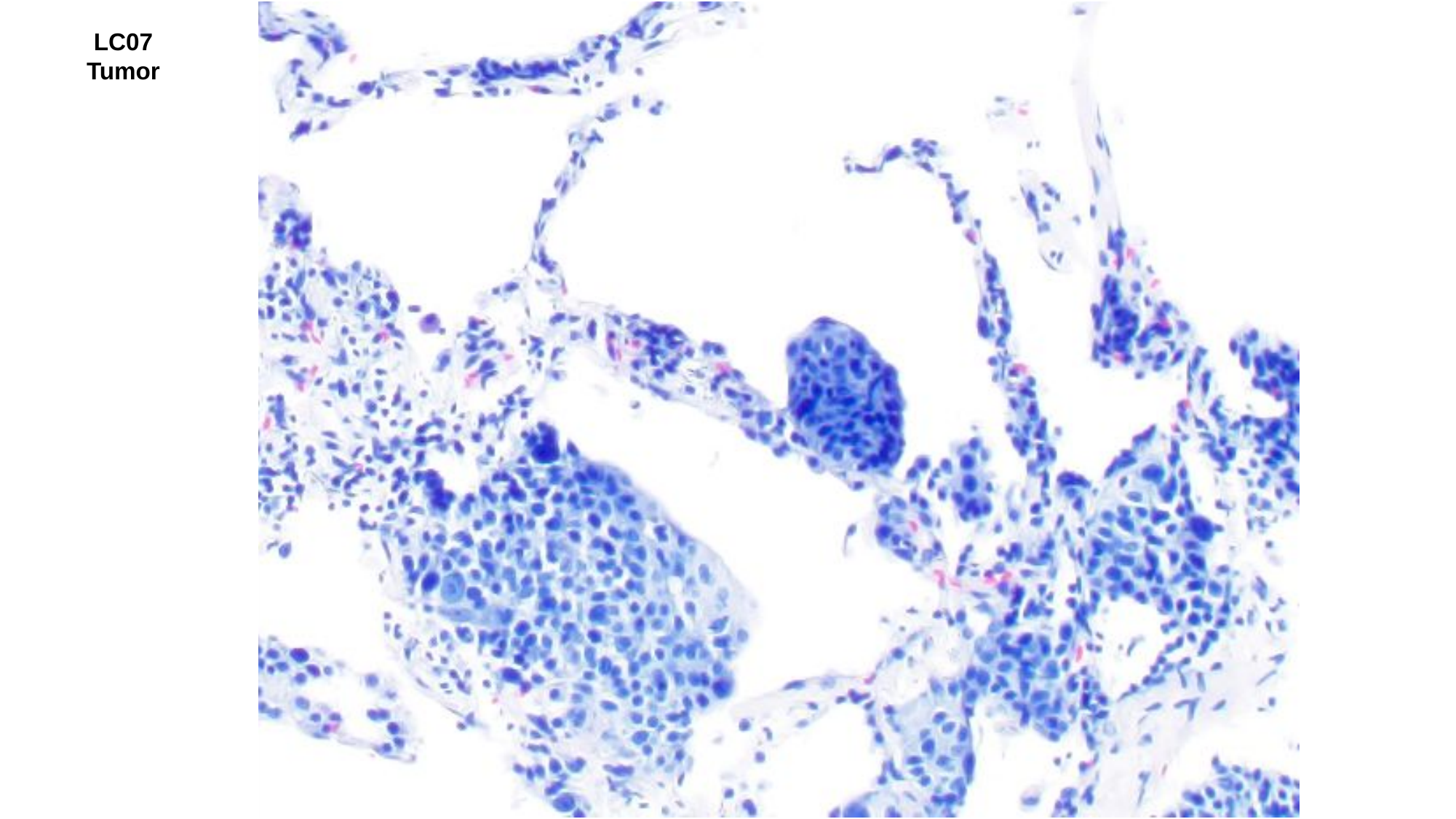

LC07
Tumor

## Slide 7
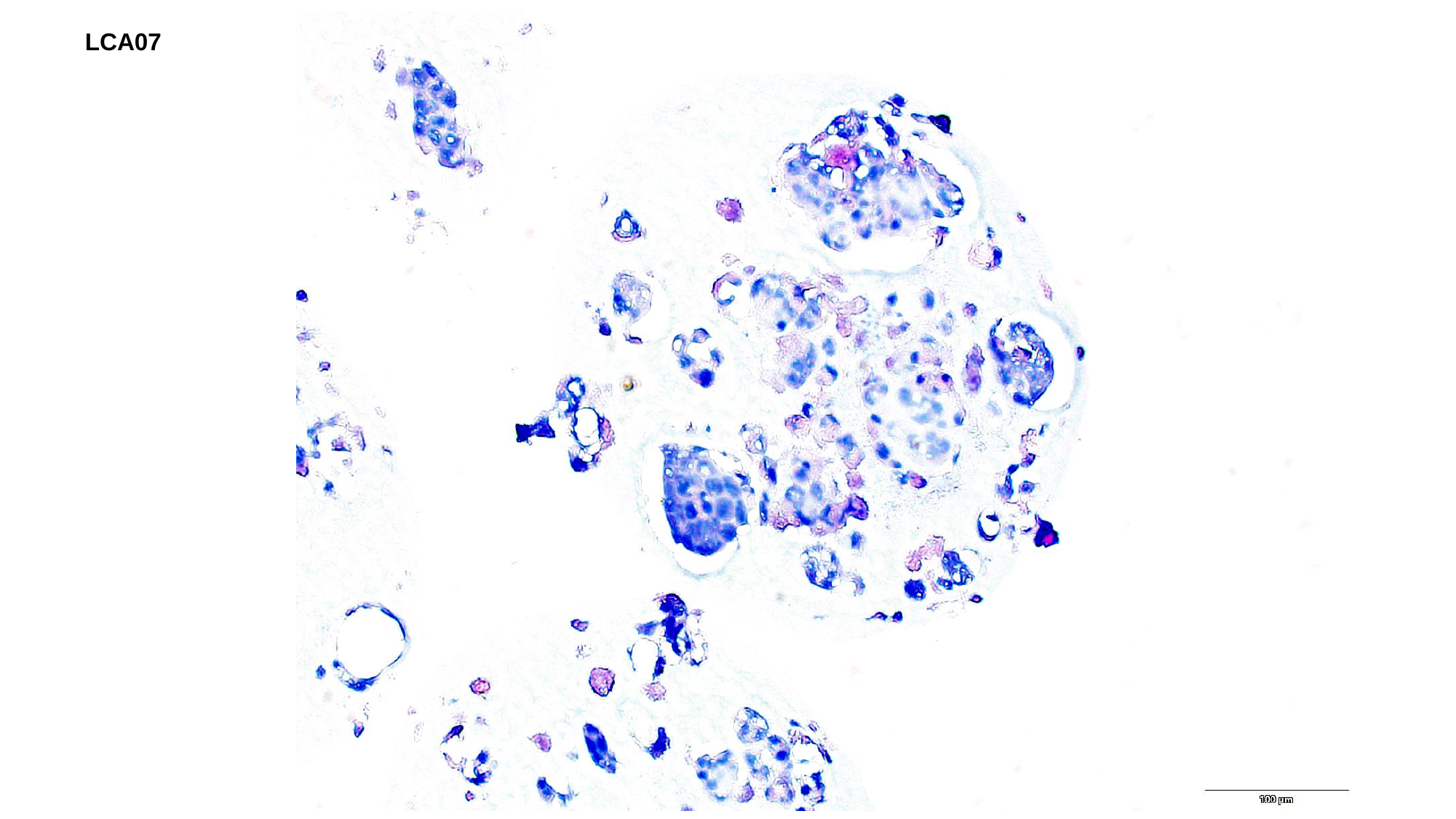

LCA07

## Slide 8
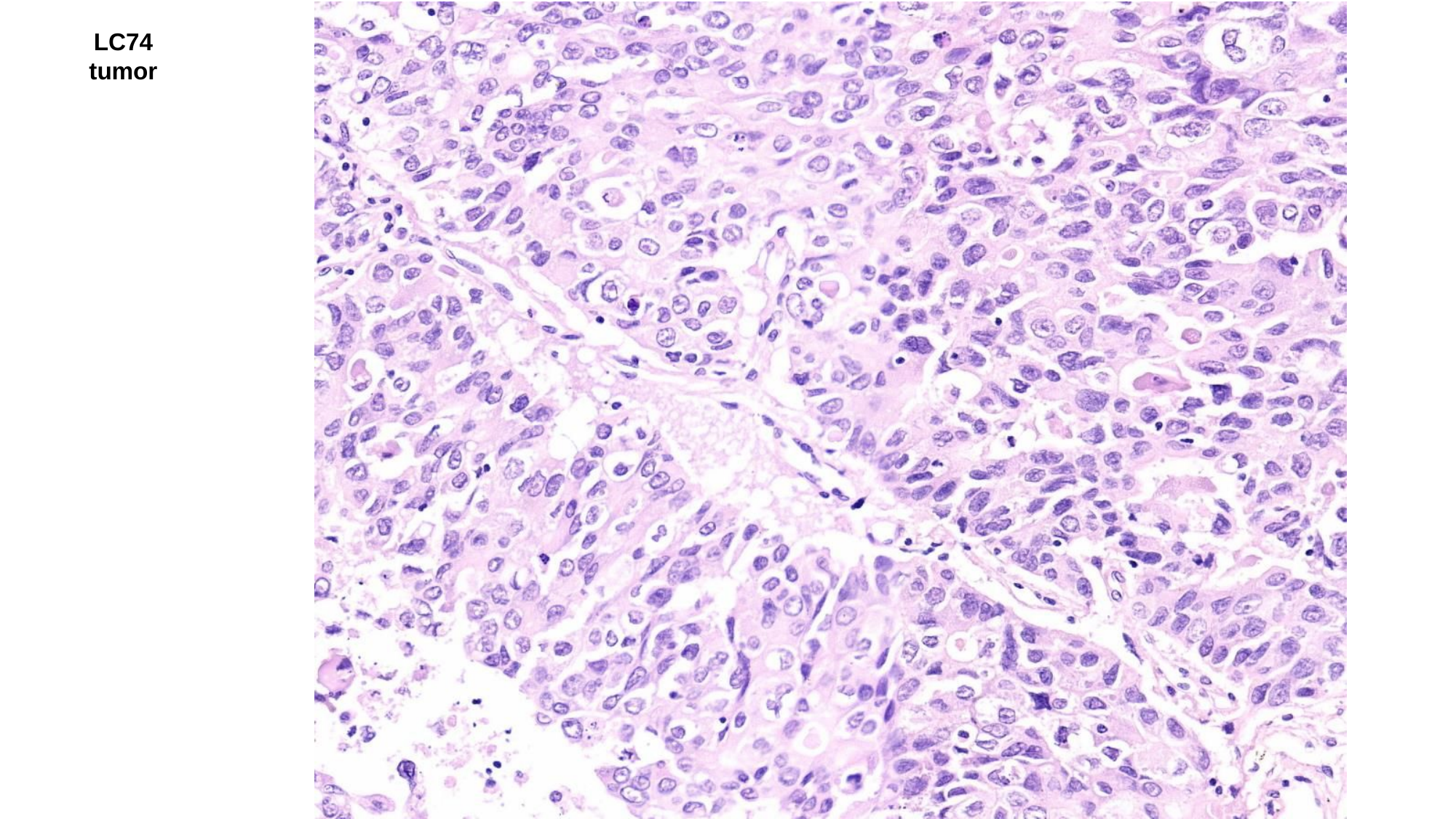

LC74
tumor

## Slide 9
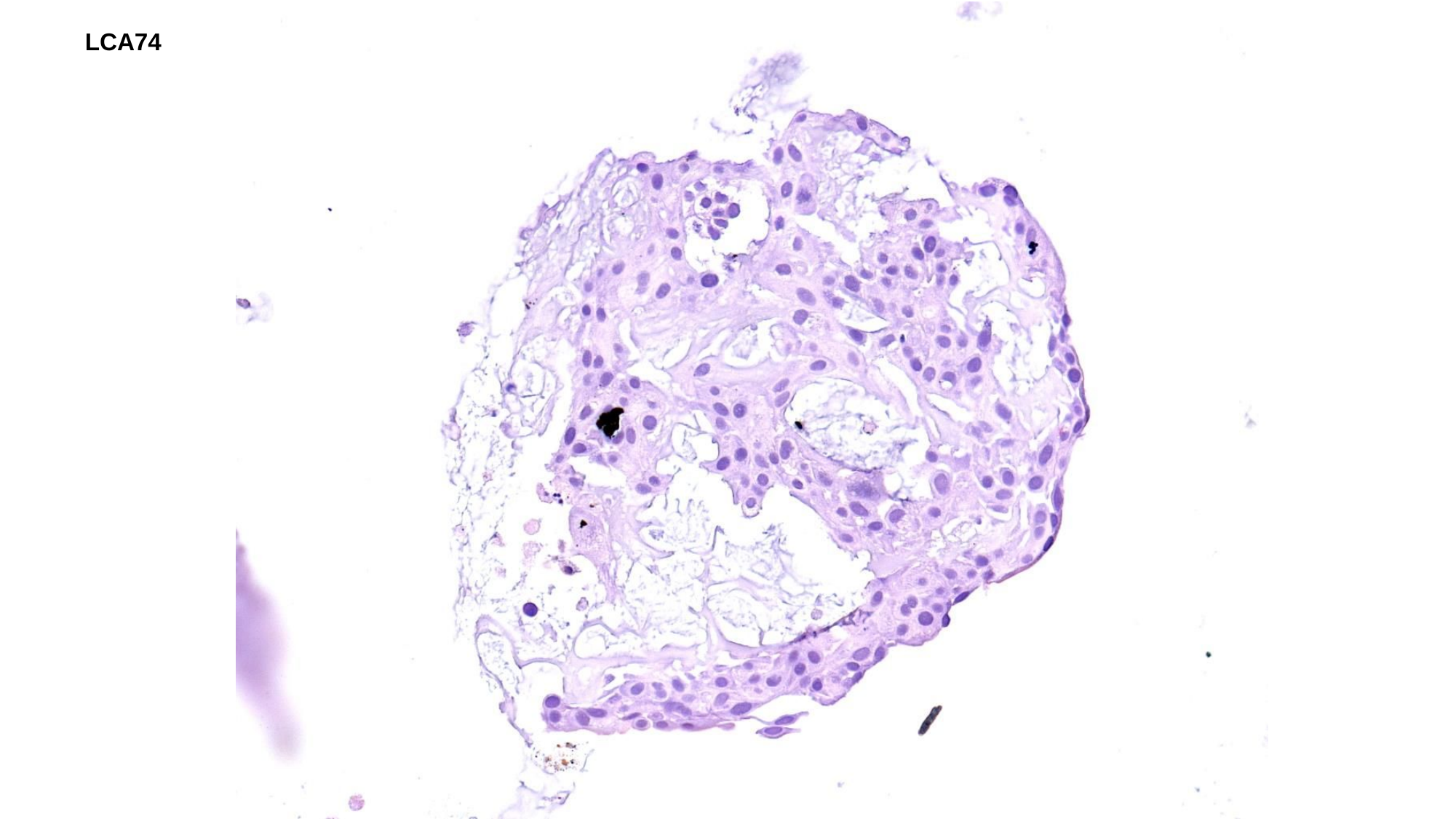

LCA74
