# Supplementary material for: A patient-specific lung cancer assembloid model with heterogeneous tumor microenvironments
Source: Nat Commun. 2024 Apr 20;15:3382. doi: 10.1038/s41467-024-47737-z (PMC11032376; doi:10.1038/s41467-024-47737-z)
Supplement: Supplementary file 5 — Reporting Summary [file 41467_2024_47737_MOESM5_ESM.pdf]

nature portfolio

Corresponding author(s): Prof. Zhuo Xiong

Last updated by author(s): Mar 3, 2024

Reporting Summary

Nature Portfolio wishes to improve the reproducibility of the work that we publish. This form provides structure for consistency and transparency in reporting. For further information on Nature Portfolio policies, see our Editorial Policies and the Editorial Policy Checklist.

Statistics

For all statistical analyses, confirm that the following items are present in the figure legend, table legend, main text, or Methods section.

n/a	Confirmed	
☐	☒	The exact sample size (n) for each experimental group/condition, given as a discrete number and unit of measurement
☐	☒	A statement on whether measurements were taken from distinct samples or whether the same sample was measured repeatedly
☐	☒	The statistical test(s) used AND whether they are one- or two-sided<br>*Only common tests should be described solely by name; describe more complex techniques in the Methods section.*
☒	☐	A description of all covariates tested
☒	☐	A description of any assumptions or corrections, such as tests of normality and adjustment for multiple comparisons
☐	☒	A full description of the statistical parameters including central tendency (e.g. means) or other basic estimates (e.g. regression coefficient) AND variation (e.g. standard deviation) or associated estimates of uncertainty (e.g. confidence intervals)
☐	☒	For null hypothesis testing, the test statistic (e.g. F, t, r) with confidence intervals, effect sizes, degrees of freedom and P value noted<br>*Give P values as exact values whenever suitable.*
☒	☐	For Bayesian analysis, information on the choice of priors and Markov chain Monte Carlo settings
☒	☐	For hierarchical and complex designs, identification of the appropriate level for tests and full reporting of outcomes
☐	☒	Estimates of effect sizes (e.g. Cohen's d, Pearson's r), indicating how they were calculated

Our web collection on statistics for biologists contains articles on many of the points above.

Software and code

Policy information about availability of computer code

Data collection	Flow cytometry data were acquired with BD LSRFortessa SORP (BD).<br>Confocal images were collected with Olympus FV3000.<br>Bright images were collected with Olympus IX73.<br>Data for histology were collected with 3D histech Pannoramic Scan (3Dhistech).<br>Data for ELISA and viability assays were collected on a SpectraMax M2 Microplate Readers (Molecular Devices).<br>Pet-CT images of patients were collected with Ingenuity CT (PHILIPS).<br>RNA seq data were collected with Illumina NovaSeq 6000

Data analysis	RNA seq and WES data were analyzed with Trim Galore (v0.6.7), BWA (v0.7.17), GATK (v4.2.2.0), ANNOVAR (v2020.06.08), CNVkit (v0.9.10),R package Maftools (2.6.05)FastQC (v0.11.5), Trimmomatic (v0.39),hisat2 (v2.1.0), HTseq (v0.13.5), R packages: limma (3.46.0), edgeR (3.32.1).<br>Sc-RNA seq data wereanalyzed with Cell Ranger (version 7.0.0),R package scCancer (v2.2.1), harmony (v1.0) and Seurat (v3.9.9).<br>Flow cytometry data were analyzed in FlowJo (v10).<br>Olympus FV31S-SW Viewer software was used to analyze confocal images.<br>The sizes or diameters were measured with Fiji image J 2.14 software.<br>The histology data were performed with CaseViewer 2.4.<br>All statistical analysis was performed with GraphPad Prism 8.0 and GraphPad Prism 9.5<br>Analysis and visualization of cell interactions were performed using the R package CellChat (1.4.0)<br>Gene set enrichment analysis (GSEA) was performed by Metascape （https://metascape.org/gp/index.html#/main/step1）

For manuscripts utilizing custom algorithms or software that are central to the research but not yet described in published literature, software must be made available to editors and reviewers. We strongly encourage code deposition in a community repository (e.g. GitHub). See the Nature Portfolio guidelines for submitting code & software for further information.

Data

Policy information about availability of data

All manuscripts must include a data availability statement. This statement should provide the following information, where applicable:

- Accession codes, unique identifiers, or web links for publicly available datasets
- A description of any restrictions on data availability
- For clinical datasets or third party data, please ensure that the statement adheres to our policy

All the raw data for the RNA-seq, exome-seq and single-cell RNA-seq data reported in this study have been deposited in GSA-Human database (Genome Sequence Archive for Human, as a part of GSA in the National Genomics Data Center) and are accessible for all readers by visiting GSA-Human [https://ngdc.cncb.ac.cn/gsa-human/] (accession numbers: HRA003470 [https://ngdc.cncb.ac.cn/gsa-human/browse/HRA003470] and HRA004052 [https://ngdc.cncb.ac.cn/gsa-human/browse/HRA004052]) under the approval of the Ministry of Science and Technology (accession number:2023BAT0242 and 2023BAT1141).The remaining data are available within the Article, Supplementary Information or Source Data file. Source data are provided with this paper.

Research involving human participants, their data, or biological material

Policy information about studies with human participants or human data. See also policy information about sex, gender (identity/presentation), and sexual orientation and race, ethnicity and racism.

Reporting on sex and gender	There is no sex and gender analysis in our manuscript.
Reporting on race, ethnicity, or other socially relevant groupings	This manuscript did not include the research about race, ethnicity or other socially constructed categories.
Population characteristics	Patients' characteristics and informations are shown in detail in Supplementary Table 1 of this manuscript.
Recruitment	Surgical excess tumors or biopsies from consented patients with confirmed diagnosis of lung cancer were included in this study. These patients were negative for infectious diseases like hepatitis-B/C,HIV and Covid-19. Contribution of infections towards response to treatment could not be evaluated in this study. Sample was selected based on the availability of the tissue sample and not biased towards any parameters.
Ethics oversight	The research protocol was approved by the Medical Institutional Review Board of Tsinghua University (accession number: 20220301). The entire experimental protocol was conducted in compliance with the institutional guidelines.

Note that full information on the approval of the study protocol must also be provided in the manuscript.

Field-specific reporting

Please select the one below that is the best fit for your research. If you are not sure, read the appropriate sections before making your selection.

- [x] Life sciences
- [ ] Behavioural & social sciences
- [ ] Ecological, evolutionary & environmental sciences

For a reference copy of the document with all sections, see nature.com/documents/nr-reporting-summary-flat.pdf

Life sciences study design

All studies must disclose on these points even when the disclosure is negative.

Sample size	The sample size is indicated in the figure legend for each experiment. The in vitro assays included at least 3 independently biological replicates.
Data exclusions	No data were excluded.
Replication	All data presented in this study had biological replicates.The sample size and number of replicates for each experiment is included in the

figure legends. Detailed information of each experiment is also provided in the Methods section.

Randomization: All samples were randomly allocated into experimental groups.

Blinding: Blinding is not relevant to this study since the samples were not devided into groups.

Reporting for specific materials, systems and methods

We require information from authors about some types of materials, experimental systems and methods used in many studies. Here, indicate whether each material, system or method listed is relevant to your study. If you are not sure if a list item applies to your research, read the appropriate section before selecting a response.

Materials & experimental systems

n/a	Involved in the study
☐	☒ Antibodies
☐	☒ Eukaryotic cell lines
☒	☐ Palaeontology and archaeology
☒	☐ Animals and other organisms
☒	☐ Clinical data
☒	☐ Dual use research of concern
☒	☐ Plants

Methods

n/a	Involved in the study
☒	☐ ChIP-seq
☐	☒ Flow cytometry
☒	☐ MRI-based neuroimaging

Antibodies

Antibodies used

Antibodies used for Immunofluorescence and IHC staining:
Rabbit monoclonal anti-EpCAM [EPR20532-225] (Abcam, ab223582, 1:1000)
Sheep polyclonal anti-vWF (Abcam, ab11713, 1:600)
Mouse anti-alpha-smooth muscle anctin antibody [1A4] (Abcam,ab7817, 1:800)
Mouse monoclonal anti-CD45 [HI30] (Biolegend, 304002,1:50)
Rabbit monoclonal CD3 antibody [SP162] (Abcam, ab135372, 1:200)
Rabbit monoclonal anti-Ki67 [EPR3610] (Abcam, ab216709, 1:1000)
Mouse monoclonal anti-E-cadherin [M168] (Abcam, ab76055, 1:200)
Rabbit monoclonal anti-FAP [E1V9V] (CST, 66562, 1:200)
Rabbit monoclonal CK7[UMAB161] (ZSGB-bio,ZM-0071, working fluid)
Alexa Fluor 647-AffiniPure Donkey Anti-Sheep IgG (H+L) (Jackson ImmunoResearch, 713-605-003, 1:500)
Alexa Fluor 488-AffiniPure Donkey Anti-rabbit IgG (H+L) (Jackson ImmunoResearch, JAC-111-545-003,1:500)
Alexa Fluor® 594-AffiniPure Goat Anti-Rabbit IgG (H+L) (Jackson ImmunoResearch,JAC-111-585-144, 1:500)
Alexa Fluor® 594-AffiniPure Goat Anti-mouse IgG (H+L) (Abcam, ab150116,1:500)

Antibodies used for Flow cytometry:
Mouse monoclonal anti-CD45 [HI30] (Biolegend, 304002,1:50)
FITC anti-human CD31 Antibody [WM59] (Biolegend 303104, 1:20)
FITC anti-human CD8a Antibody[RPA-T8](Biolegend, 301006, 1:20)
Rabbit monoclonal anti-EpCAM [EPR20532-225] (Abcam, ab223582, 1:500)
The antibody informations are shown in detail in Supplementary Table 7 of this manuscript.

Validation

All commercial antibodies were verified by the supplier and each lot has been quality tested. Validation data are available on the manufacturer's website. And all the primary antibodies were optimized using positive tissues as recommended by the manufacturers.
Antibodies used for Immunofluorescence staining:
Rabbit monoclonal anti-EpCAM [EPR20532-225] (Abcam, ab223582);https://www.abcam.cn/products/primary-antibodies/epcam-antibody-epr20532-225-ab223582.html
Sheep polyclonal anti-vWF (Abcam, ab11713) https://www.abcam.cn/products/primary-antibodies/von-willebrand-factor-antibody-ab11713.html
Mouse anti-alpha-smooth muscle anctin antibody [1A4] (Abcam,ab7817);https://www.abcam.cn/products/primary-antibodies/alpha-smooth-muscle-actin-antibody-1a4-ab7817.html
Mouse monoclonal anti-CD45 [HI30] (Biolegend, 304002); https://www.biolegend.com/en-gb/products/purified-anti-human-cd45-antibody-710?GroupID=BLG5926
Rabbit monoclonal CD3 antibody [SP162] (Abcam, ab135372);https://www.abcam.cn/products/primary-antibodies/cd3-antibody-sp162-ab135372.html
Rabbit monoclonal anti-Ki67 [EPR3610] (Abcam, ab216709); https://www.abcam.cn/products/primary-antibodies/alexa-fluor-594-ki67-antibody-epr3610-ab216709.html
Mouse monoclonal anti-E-cadherin [M168] (Abcam, ab76055);https://www.abcam.cn/products/primary-antibodies/e-cadherin-antibody-m168-c-terminal-ab76055.html
Rabbit monoclonal anti-FAP [E1V9V] (CST, 66562);https://www.cellsignal.cn/products/primary-antibodies/fap-e1v9v-rabbit-mab/66562

Rabbit monoclonal CK7[UMAB161] (ZSGB-bio,ZM-0071, working fluid);http://www.zsbio.com/product/ZM-0071

Alexa Fluor 647-AffiniPure Donkey Anti-Sheep IgG (H+L) (Jackson ImmunoResearch, 713-605-003, 1:500);https://www.jacksonimmuno.com/catalog/products/713-605-003#collapseTwo
Alexa Fluor 488-AffiniPure Donkey Anti-rabbit IgG (H+L) (Jackson ImmunoResearch, JAC-111-545-003,1:500)https://www.jacksonimmuno.com/catalog/products/111-545-003#collapseFour
Alexa Fluor® 594-AffiniPure Goat Anti-Rabbit IgG (H+L) (Jackson ImmunoResearch,JAC-111-585-144, 1:500)https://www.jacksonimmuno.com/catalog/products/111-585-144#collapseFour
Alexa Fluor® 594-AffiniPure Goat Anti-mouse IgG (H+L) (Abcam, ab150116,1:500);https://www.abcam.cn/products/secondary-antibodies/goat-mouse-igg-hl-alexa-fluor-594-ab150116.html

Antibodies used for Flow cytometry:
Mouse monoclonal anti-CD45 [HI30] (Biolegend, 304002,1:50); https://www.biolegend.com/en-gb/products/purified-anti-human-cd45-antibody-710?GroupID=BLG5926
FITC anti-human CD31 Antibody [WM59] (Biolegend 303104, 1:20); https://www.biolegend.com/en-gb/products/fitc-anti-human-cd31-antibody-881
FITC anti-human CD8a Antibody[RPA-T8](Biolegend, 301006, 1:20);https://www.biolegend.com/en-gb/products/fitc-anti-human-cd8a-antibody-834
Rabbit monoclonal anti-EpCAM [EPR20532-225] (Abcam, ab223582, 1:500);https://www.abcam.cn/products/primary-antibodies/epcam-antibody-epr20532-225-ab223582.html

Eukaryotic cell lines

Policy information about cell lines and Sex and Gender in Research

Cell line source(s)	The cell line only used in this manuscript is A549 cell line obtained from the American Type Culture Collection (ATCC).
Authentication	Upon receipt from the supplier, the cell line was expanded and immediately cryopreserved. For each new experiment, fresh cells were thawed from liquid nitrogen tank. The Cell line was authenticated by periodic morphology check via microscope.
Mycoplasma contamination	The A549 cell line was tested for mycoplasma contamination. No mycoplasma contamination was found.
Commonly misidentified lines (See ICLAC register)	No commonly misidentified cell lines are used in this study.

Plants

Seed stocks	Not involve
Novel plant genotypes	Not involve
Authentication	Not involve

Flow Cytometry

Plots

Confirm that:

- [x] The axis labels state the marker and fluorochrome used (e.g. CD4-FITC).
- [x] The axis scales are clearly visible. Include numbers along axes only for bottom left plot of group (a 'group' is an analysis of identical markers).
- [x] All plots are contour plots with outliers or pseudocolor plots.
- [x] A numerical value for number of cells or percentage (with statistics) is provided.

Methodology

Sample preparation	Cell apoptosis analysis in LCA was performed by using Annexin V-FITC Apoptosis Detection Kit (YEASEN, China) according to the manufacturer's instructions. In brief, LCAs were dissociated with trypsin containing 0.25% EDTA (Sigma) and washed in PBS buffer. 2 ×105 cells were collected and resuspended in Annexin V binding buffer (200 μL), Annexin V-FITC (5 μL), PI (5 μL) and anti-human EpCAM-APC (5 μL, marking the tumor cells in LCAs) were added. The cells were incubated at room temperature for 15 min in the dark and then resuspended in binding buffer (300 μL) after centrifugation. Finally, cell apoptosis was analyzed via flow cytometry (BD LSRFortessa, BD Biosciences).
Instrument	BD LSRFortessa (BD Biosciences)
Software	Flow cytometry data were analyzed in FlowJo (v10).
Cell population abundance	A cell count of 10,000 events was collected for relevant cell populations after initial gating.
Gating strategy	Initial cell populations were gated for singlet and doublet cells using FSC-A/ FSC-H and SSC-A/SSC-H gating. Parental cells without staining were used to set the background staining gates. The EpCAM/E-cadherin positive populations were performed further apoptosis analysis.

☒ Tick this box to confirm that a figure exemplifying the gating strategy is provided in the Supplementary Information.
